# 

**Published:** 1972-07

**Authors:** 


The
Bristol Medico-Chirurgical Journal
(Incorporating the Medical Journal of the South-West)
Established 1883

Vol. 87 (iii) JULY 1972 No. 323

### BRISTOL MEDICO-CHIRURGICAL JOURNAL

Editor: N. J. Brown, M.B., F.R.C.P., F.R.C.Path.

Published Quarterly

Annual Subscription £2 Post Free

### BRISTOL MEDICO-CHIRURGICAL SOCIETY

**President 1971-72 : Dr. C. C. Morgans**

**President-elect : Dr. James Macrae.**

**Honorary Secretary : Dr. I. S. Bailey, 7 Percival Road, Bristol 8.**

**Honorary Treasurer : Dr. Frank Ross, Bristol Royal Infirmary, Bristol 2.**

The Society was founded in 1874. In 1883 publication of the Journal began and has continued quarterly ever since then. During the years 1949 to 1962 it appeared under the title of "The Medical Journal of the South West".

### THE BRISTOL MEDICO-CHIRURGICAL JOURNAL

**Editorial Committee**

Editor Dr. N. J. Brown, Southmead Hospital, Bristol

Members Dr. Rhys Davies.
Dr. M. B. Lennard.
Professor R. C. Wofinden.
Dr. D. W. Wright.
The Hon. Treasurer and Hon. Secretary of the Society.

Business Manager Dr. P. J. F. Baskett, Frenchay Hospital, Bristol.

Secretary Mr. B. P. Jones, The Library, Medical School, University Walk, Bristol BS8 1TD.

### NOTICE TO CONTRIBUTORS

The Journal is especially concerned to provide a place for the recording of medical thought, interests, and practice in and around Bristol. It therefore welcomes articles that, as well as being of immediate interest to members, will document the local and contemporary medical scene for our successors.

Original articles are invited provided that they have not been published elsewhere. References in the text should be by author's name, with year of publication in parenthesis; they should be listed at the end in alphabetical order, the name of each journal or book being given in full—not abbreviated—and the title of the article added. Illustrations should be supplied ready for reproduction. Line drawing should be in Indian ink on firm white paper or board. The author will be informed if it is found necessary to ask him to pay for the making of blocks.

The following contributions will be especially welcome; notes of forthcoming events, meetings held, degrees conferred, staff appointments, honours and public appointments and other local news.

Twenty-five reprints of his article will be supplied free to each contributor. Quotations for larger quantities will be sent out with proofs and must be ordered when the corrected proofs are returned.

### Appendix

### PUBLISHED WORKS BY THE HOSPITAL STAFF

(A) BOOKS

**1952**

(1) **Lyons, J. F., and Heaton-Ward, W. A.**
Notes on Mental Deficiency. John Wright & Sons Ltd. Bristol (Third Edition 1955).

**1957**

(2) **Woods, Grace E.**
Cerebral Palsy in Childhood. John Wright & Sons Ltd. Bristol.

**1958**

(3) **Hallas, C. H.**
Nursing the Mentally Subnormal. John Wright & Sons Ltd. Bristol. (Second Edition 1962).

**1968**

(4) **Eastham, R. D. and Jancar, J.**
Clinical Pathology in Mental Retardation. John Wright & Sons Ltd. Bristol.

(B) PAPERS

**1932**

(1) **Rudolf. G. de M.**
Industries in Mental Deficiency Colonies. Final Rep. Public Health Cong. and Exhibition, 1932, p.90.

**1933**

(2) **Rudolf, G. de M.**
The Mental Deficiency Institution in the Prevention of Crime. J. Ment. Sc. **79**, 554.

**1934**

(3) **Rudolf, G. de M.**
The Value of the Treatment of Mental Deficiency. Proc. Roy. Soc. Med. **27**, 1742.

(4) **Rudolf, G. de M.**
Diagnosis and Grading of Oligophrenia, Rep. 11th Assembly Internat. Fed. Eugenic Organiz. Zurich, p.15.

**1935**

(5) **Rudolf, G. de M.**
Mental Defects from the Neurological and Psychiatric Standpoints. Proc. Roy. Soc. Med. **28**, 800.

**1938**

(6) **Rudolf, G. de M.**
Delinquency in the Mentally Defective. Mental Hygiene. **4**, 82.

**1941**

(7) **Rudolf, G. de M.**
The Development of Knowledge in the Mental Defective based on Kent's Oral test. Brit. J. Med. Psychol. **18**, 338.

**1947**

(8) **Rudolf, G. de M.**
Treatment Rep. Mental Defic. Acts committee, Bristol. p.6.

**1948**

(9) **Rudolf, G. de M.**
The Kent and other Tests used on the same subjects. J. Ment. Sc., **94**, 452.

(10) **Rudolf, G. de M.**
The Ephedrine Treatment of Nocturnal Enuresis. J. ment. Sc., **94**, 629.

**1949**

(11) **Rudolf, G. de M.**
Re-testing of the Intelligence Quotient and the Social Age. J. Ment. Sc. **95**, 696.

(12) **Rudolf, G. de M.**
Comparison of the Intelligence Quotient with Behaviour. J. Ment. Sci. **95**, 703.

(13) **Rudolf, G. de M.**
The Treatment of Mental Defectives with Thiamine. J. ment. Sc. **95**, 910.

**1950**

(14) **Rudolf, G. de M.**
The Treatment of Mental Defective with Aneurin for one year. J. Ment. Sci. **96**, 265.

(15) **Rudolf, G. de M.**
Improvement of Mental Defectives in Colonies. J. ment. Sci. **96**, 272.

(16) **Rudolf, G. de M.**
Changes continuing after the termination of Treatment of Mental Defectives with Aneurin. J. ment. Sci. **96**, 769.

(17) **Rudolf, G. de M.**
The Psycho-Therapist and Extra-sensory Perception. Brit. J. Med. Psych. **23**, 105.

**1951**

(18) **Rudolf, G. de M.**
Responsibility in English Law. Med. Press. p.573.

**1952**

(19) **Rudolf, G. de M.**
The Effect of Sex Hormones in Oligophrenia. J. ment. Sci. **98**, 294.

**1954**

(20) **Rudolf, G. de M.**
An Experiment in the Treatment of Masturbation in Oligophrenia. Amer. J. Ment. Defic. **58**, 644.

(21) **Rudolf, G. de M. and Ingleby, A. H. B.**
Protozoal behaviour as a possible cause of tumour formation. Med. Press, 231.

(22) **Rudolf, G. de M.**
Homosexuality. Lancet, Oct. 2nd.

**1955**

(23) **Rudolf, G. de M.**
An Experiment in Group Therapy with Mental Defectives. Internat. J. Soc. Psychiat. **1**, 49.

(24) **Rudolf, G. de M.**
Innocuous temporary immobility. Lancet. **1**, 51.

(25) **Rudolf, G. de M.**
The treatment of depression with sympathomimetic preparations. Practitioner. **174**, 180.

(26) **Rudolf, G. de M.**
An investigation into the cinematograph and deterioration in behaviour. Mental Health, **14**, 50.

(27) **Rudolf, G. de M.**
Innocuous temporary immobility. Lancet. **1**, 675.

(28) **Rudolf, G. de M.**
Drugs in the treatment of Depression. Lancet. **1**, 1178.

(29) **Rudolf, G. de M.**
The Jury-Group. Medico-Legal J. **23**, 12.

(30) **Rudolf, G. de M.**
The effect of Primidone (Mysoline) on Grand Mal attacks. Dis. Nerv. Syst. **16**, 204.

(31) **Rudolf, G. de M.**
Psychiatry and the Marriage Guidance Counsellor. Marr. Guid. Bull. **9**, 9.

(32) **Rudolf, G. de M.**
Clinical Blood Pressure in Anxiety. J. ment. Sci. **101**, 893.

(33) **Rudolf, G. de M.**
Submarine Crews. Minutes Members Mtg. Roy. Coll. Physicians. May 5th, p.8.

(34) **Rudolf, G. de M.**
Length of sleep in adults. Med. Press. **233**, 324.

**1956**

(35) **Rudolf, G. de M.**
Amphetamine Preparations in Psychiatry. Med. Illus. **10**, 106.

(36) **Rudolf, G. de M.**
Cinematograph and Behaviour. Ment. Health, **15**, 75.

(37) **Rudolf, G. de M.**
A Tonic Immobility. Practit. **176**, 537.

(38) **Rudolf, G. de M.**
The treatment of Depression with Methylapheta-mine. J. ment. Sci. **102**, 358.

(39) **Rudolf, G. de M.**
Preparation for Marriage. Brit. Med. J. **2**, 244.

**1958**

(40) **Walker, W. L.**
Problems in the Differential Diagnosis of Mental Defect in Childhood. J. Mid. Men. Defic. Soc. **4**, 42.

(41) **Rudolf, G. de M.**
The Effect of Children's Television on Behaviour. Mental Health. **17**, 50.

(42) **Woods, Grace E.**
The Early Diagnosis of Infantile Cerebral Palsy. Pub. Health.

(43) **Norman, R. M., Urich, H., and Woods, Grace E.**
The Relationship between Prenatal Porencephaly and the Encephalomalacias of Early Life. J. ment. Sci. **104**, 758.

(44) **Woods, Grace E.**
The Development of Body Image. Cerebral Palsy Bulletin.

**1959**

(45) **Walker, W. L.**
Return to the Community. J. Mid. Men. Defic. Soc. **5**, 1.

(46) **Woods, Grace E.**
Infantile Cerebral Palsy. Helping the Parents. Medical World. **91**, 225.

**1960**

(47) **Rudolf, G. de M.**
Success and Failure on leave. J. ment. Subnorm. **6**, 73.

(48) **Woods, Grace E.**
Clinical Problems affecting the education of the cerebral-palsied child. British Council for Welfare of Spastics.

(49) **Woods, Grace E.**
The Early Diagnosis of Cerebral Palsy. Cerebral Palsy Review, Kansas, U.S.A.

(50) **Illingworth, R. S. and Woods, Grace E.**
The Incidence of Twins in Cerebral Palsy and Mental Retardation. Arch. Dis. Childh. **35**, 333.

**1961**

(51) **Buckton, K. E., Harnden, D. G., Baikie, A. G. and Woods, Grace E.**
Mongolism and Leukaemia in the same Sibship. Lancet. **1**, 171.

(52) **Rudolf, G. de M.**
Crime amongst the unsupervised mentally subnormal. Medical Press. **245**, 466.

(53) **Rudolf, G. de M.**
Deconditioning and Time-Therapy. J. ment. Sci. **107**, 1097.

(54) **Woods, Grace E.**
The Ineducable Spastic, Spastics Quarterly.

**1962**

(55) **Jancar, J.**
Mandibulo-Facial Dysostosis (Berry-Franceschetti Syndrome) associated with Severe Mental Subnormality and Consanguinity. Proc. London Conf. Scientific Study Mental Deficiency, London. **1**, 329.

(56) **Jancar, J.**
Melleril and Placebo in the Treatment of Severely Subnormal Patients. J. ment. Subnorm. **8**, 52.

(57) **Woods, Grace E.**
Preliminary Clinical Trial of Carisoprodal. Develop. Med. Child. Neurol. **4**, 28.

(58) **Woods, Grace E.**
Double Blind Trial of Carisoprodal. Develop. Med. Child. Neurol. **4**, 499.

**1963**

(59) **Brown, R. I. and Clarke, A. D. B.**
The Effects of Auditory Distraction on Institutionalised Subnormal and Severely Subnormal Persons. J. ment. Defic. Res. **7**, 1.

(60) **Walker, W. L.**
Intellectual and Behavioural consequences of Prematurity with Birth Asphyxia. Aberdeen. University thesis.

(61) **Rudolf, G. de M.**
The treatment of spasticity with Chlordiazepoxide (Librium). Brit. J. Psychiat. **109**, 544.

(62) **Rudolf, G. de M.**
Last Conscious Thoughts. Bristol Med-chir. P. **78**, 125.

(63) **Jancar, J.**
Rickets with Secondary Hyperparathyroidism in a Severely Subnormal Child. Arch. Dis. Childh. **38**, 412.

(64) **Woods, Grace E.**
A Lowered Incidence of Infantile Cerebral Palsy. Develop. Med. Child Neurol. **5**, 449.

**1964**

(65) **Brown, R. I.**
The effect of Visual Distraction on Perception in Subjects of Subnormal Intelligence. Brit. J. Soc. Clin. Psychol. **2**, 20.

(66) **Brown, R. I.**
Distraction in Subnormals. Proc. Int. Copenhagen Congr. on the Scientific Study of Mental Retardation. **1**, 351.

(67) **Jancar, J.**
Mentally Defective Males with XXXXY Chromosomes. Proc. Int. Copenhagan Congr. for the Scientific Study of Mental Retardation. **1**, 179.

(68) **Lewis, F. J. W. and Jancar, J.**
Presumptive Translocation between a "21" Chromosome and one of the 6-12 + X Group. The Human Chromosome. Newsletter **12**, 9.

(69) **Walker, W. L.**
Premature Children with Birth Asphyxia. Develop. Med. Child. Neurol. **5**, 458.

(70) **Wethered, R. R.**
Herpes Zoster Generalizatus. The Practitioner. **193**, 798.

(72) **Woods, Grace E.**
The Outcome of Physical Treatment in Cerebral Palsy. Cerebral Palsy Review. **25**, 5.

(72) **Woods, Grace E. and Walters, Ruth M.**
Lead Poisoning in Mentally Subnormal Children. Lancet, **2**. 592.

(73) **Galliford, D., James, F. E. and Woods, Grace E.**
Laterality in Athetoid Cerebral palsied Children. Develop. Med. Child. Neurol. **6**, 261.

**1965**

(74) **Eastham, R. D. and Jancar, J.**
Plasma Viscosity in cases of Severe Mental Subnormality. Amer. J. ment. Defic. **69**, 502.

(75) **Jancar, J.**
Cerebro - Metacarpo - Metatarsal Dystrophy (Pseudo-Pseudo-Hypoparathyroidism) with Chromosomal Anomaly. J. med. Genet, **2**, 32.

(76) **Jancar, J.**
The use of Haloperidol in the Treatment of Severe Behaviour Disorders in Mental Deficiency. Clin. Trials J. **2**, 154.

(77) **Jancar, J. and Philpot, G. R.**
Porphobilinogen-like Chromogens in Urine of Epileptics. Brit. med. J. **1**, 1498.

(78) **Eastham, R. D., Jancar, J. and Duncan, E. H. L.**
Plasma Viscosity in Mental Deficiency and Down's Syndrome. Brit. J. Psychiat. **111**, 999.

(79) **Jancar, J.**
Rubinstein-Taybi's Syndrome. J. met. Defic. Res. **9**, 266.

(80) **Woods, Grace E.**
Some Clinical Notes and Electro-encephalographic findings in Cerebral Palsy. Arch. Dis. Child. **40**, 394.

**1966**

(81) **Jancar, J.**
Inverse Jaw-Winking with Exaggerated Bell's Phenomenon ("Ocular Stammer"). Opthalmologica, Basel, **151**, 548.

(82) **Jancar, J.**
Hallerman-Streiff-Francois Syndrome (Dysephalia Mandibulo-Oculo-Facialis). J. ment. Def. Res. **10**, 255.

**1967**

(83) **English, Mary P., Wethered, R.R. and Duncan, Ethel, H. L.**
Studies in the Epidemiology of Tinea Pedis VIII. Fungal Infection in a long stay hospital. Brit. Med. J. **3**, 136.

(84) **Jancar, J.**
Ectrodactyly Spastic Paraplegia and Mental Retardation. J. ment. Defic, Res. **11**, 207.

(85) **Jancar, J.**
Naevus Syringocystadenomatosus Papilliferus. Proc. 2nd Int. Congr. of Neuro-Genetics and Neuro-Ophthalmology of The World Federation of Neurology. Montreal, Excerpta Medica No. 154. p.85.

(86) **Wethered, R.R., Heeler, W.R. and Warin, R. P.**
Clinical Trials in Tolmaftate and Variontin. Brit. J. Derm. **79**, 352.

**1968**

(87) **Eastham, R. D. and Jancar, J.**
Serum Cholesterol in Mental Retardation. Proc. 1st Congr. of Inst. Ass. for the Scientific Study of Mental Deficiency, Montpellier. p.948.

(88) **Jancar, J.**
XXYY with Manic Depression. Lancet, **2**, 970.

**1970**

(89) **Fairburn, A.C.**
Disturbed Families. Proc. of the Meeting of the National Society for Infant and Child Welfare, London.

